# Novel Wideband MIMO Antennas That Can Cover the Whole LTE Spectrum in Handsets and Portable Computers

**DOI:** 10.1155/2014/694805

**Published:** 2014-01-16

**Authors:** Mohamed Sanad, Noha Hassan

**Affiliations:** ^1^Amant Antennas, Lot 13/C, Second Industrial Zone, 6 October City, Giza 12451, Egypt; ^2^Faculty of Engineering, Cairo University, Giza, Egypt

## Abstract

A dual resonant antenna configuration is developed for multistandard multifunction mobile handsets and portable computers. Only two wideband resonant antennas can cover most of the LTE spectrums in portable communication equipment. The bandwidth that can be covered by each antenna exceeds 70% without using any matching or tuning circuits, with efficiencies that reach 80%. Thus, a dual configuration of them is capable of covering up to 39 LTE (4G) bands besides the existing 2G and 3G bands. 2 × 2 MIMO configurations have been also developed for the two wideband antennas with a maximum isolation and a minimum correlation coefficient between the primary and the diversity antennas.

## 1. Introduction

Customers' increasing expectations for speed, bandwidth, and global access are driving the evolution of wireless broadband technology. Customers want more information such as business, consumer applications, and entertainment available through their mobile devices, but with greater speeds [1]. LTE represents the next big step towards the 4th generation (4G) of radio technologies which is expected to increase the capacity and the speed of mobile telephone networks. These expectations put a significant burden on device performance. The lack of spectrum harmonization represents a key challenge for the emerging LTE ecosystem, potentially preventing vendors from delivering globally compatible LTE products such as devices and chipsets [2]. Spectrum fragmentation has the potential to hinder global LTE roaming if device manufacturers are required to include support for many disparate frequencies in their devices. Yet, in fragmented regional markets such as Europe, LTE roaming is some way off as operators will be providing LTE in different bands and the devices will need to be able to seamlessly switch between the frequency bands used for LTE in addition to the 2G and 3G networks [3].

### 1.1. Conventional Antenna Challenges in LTE

The antenna is becoming an increasingly critical component for LTE device vendors. It is possible that future terminal devices will have more than 20 antennas to cover all the important wireless applications [4]. In addition, the industry is painfully aware of the issues that surround implementing LTE in small mobile devices with already limited space and extremely high performance expectations. Due to this trend, wideband antenna coverage is a hot issue that has to be addressed [5]. Device makers are finding it difficult to decide which bands to prioritize as chipsets and handsets are developed. The priority would be for the 800 MHz band. Most of the investments has been directed towards that band because it is the band that two-thirds of current LTE users occupy, largely driven by the U.S. network roll outs of Verizon Wireless and AT&T [3]. This low frequency band means larger antennas in terms of size, which is a challenging issue, keeping in mind the limited size of LTE devices. The urgent demand for wideband LTE coverage represents a serious challenge facing antenna designers. Adapting the conventional antenna technology to serve the wideband demand was not a much of a success, leading to a definitive belief that passive antennas have reached their limits [6]. Thus, the active tuneable antenna approach has been adopted to fulfil the market need for world LTE devices, trading the passive antenna simplicity with active antenna complexity.

### 1.2. Disadvantages of Active Antennas and Extended Grounds Planes

Passive antennas have various advantages over the active tuneable antennas. First of all, the passive antenna does not have to be supported with RF controlling circuit to do the job as the active one, so it will save a large space required inside the handset to fit both the antenna and the RF circuit. This is not the only problem of the RF circuit, as additional circuit components are connected to the antenna; it will suffer from impedance mismatch. In this situation a severe decrease in the efficiency of the antenna will lead to a lower speed of data transfer and a higher number of dropped calls. Also, the active antenna is power consuming, causing a significant decrease in the battery lifetime. Thus, it will be a problem for power hungry, smart handheld devices. Last but not least, the active antenna seems to have band limitations too, as the maximum number of bands that commercial LTE active antennas can support is 13 bands only out of 39 potential LTE bands [6]. On the other hand, current handset antenna technologies are still tied with the extended ground plane's dilemma. As the antenna is not just the module that fits under the speaker, however, it includes a large PCB ground plane. A minimum limit size of the ground plane is required for the antenna to have an acceptable performance. Thus, adding an extravolume counted on the total size of the antenna. As the ground plane is a part of the antenna structure, then, the hand grip on the phone will cause detuning of the antenna operating frequency causing a poor efficiency.

### 1.3. A Novel Resonant Wideband Passive Antenna

**Figure 1:**
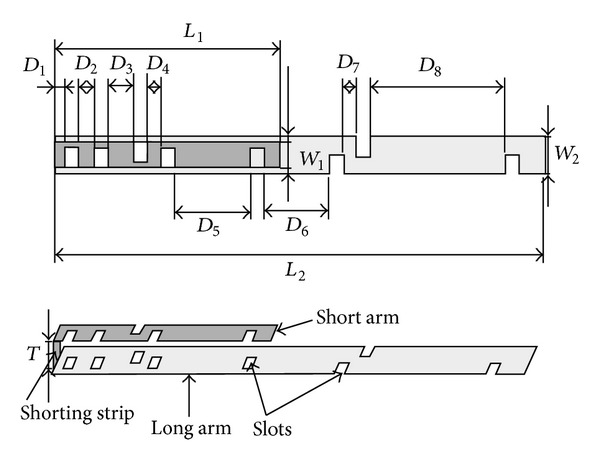
Geometry of the new wideband antenna.

A novel antenna technology has been developed to solve the problem of the urgent need for universal LTE devices. The new technology can cover all the possible LTE spectrum bands using only two antennas with bandwidths of 73% and 85%, respectively, without using any matching or tuning circuits. The first antenna is covering the low band LTE spectrum starting from 698 MHz up to 1.51 GHz which includes LTE band numbers 5, 6, 8, 11, 12, 13, 14, 17, 18, 19, 20, 21, 26, 27, 28, 29, and 44. The second one is covering the high band portion of the LTE spectrum starting from 1.52 GHz to 3.8 GHz corresponding to LTE band numbers 1, 2, 3, 4, 7, 9, 10, 22, 23, 24, 25, 33, 34, 35, 36, 37, 38, 39, 40, 41, 42, and 43. This means that a total number of 39 LTE bands can be covered beside the 2G and 3G frequency bands. The new antenna can be implemented in smart phone handsets, tablets, laptops, and notebooks. It consists of two narrow printed metallic arms connected together by a shorting metallic strip. The two arms may be parallel to each other or may have any angle between them. The two arms can be shaped in different ways in order to optimize the antenna performance. As shown in Figure 1, each arm has a set of slots having different configurations. These slots can be circular, rectangular, square, triangular, or other shapes. The arm lengths of the new antenna, especially the length of the short arm, are the main parameters that determine the operating frequency of the antenna. The bandwidth, the peak gain, and the efficiency of the antenna are mainly determined by the widths of the two arms, the angle between them, the thickness of the antenna, and the configurations of the slots, which are all optimized together in order to enhance the antenna performance, especially the bandwidth. This geometry can be scaled and optimized for any application to successfully cover any frequency band [7, 8].

## 2. Numerical and Experimental Results of the New Antenna

Two versions of the new LTE antenna have been designed, manufactured, and tested. Calculated and measured return losses will be presented in addition to the calculated gain, efficiency, and radiation patterns. The first antenna version can cover a frequency band from 698 MHz up to 1.51 GHz and it will be referred to as “the low band antenna.” To cover the high band portion of the LTE spectrum, a second version of the antenna has been designed and manufactured by scaling and optimizing the geometry shown in Figure 1. The antenna is operating all over the high frequency band 1.52–3.8 GHz and it will be referred to as “the high band antenna.” A reduced size version of these wideband antennas can be developed and customized for smaller LTE devices. Together, the two reduced size antennas are capable of covering 38 LTE bands with slightly lower but still acceptable efficiencies. The reduced size versions of the low band and high band antennas will be also demonstrated.

### 2.1. The Low Band and High Band Antennas

**Figure 2:**
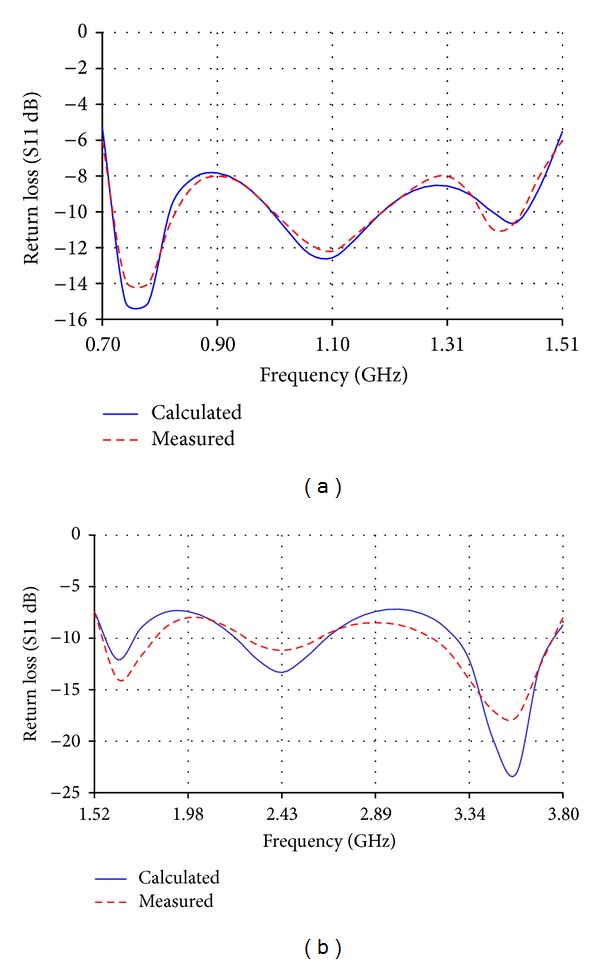
Return loss (S11) of (a) the low band antenna and (b) the high band antenna.

**Figure 3:**
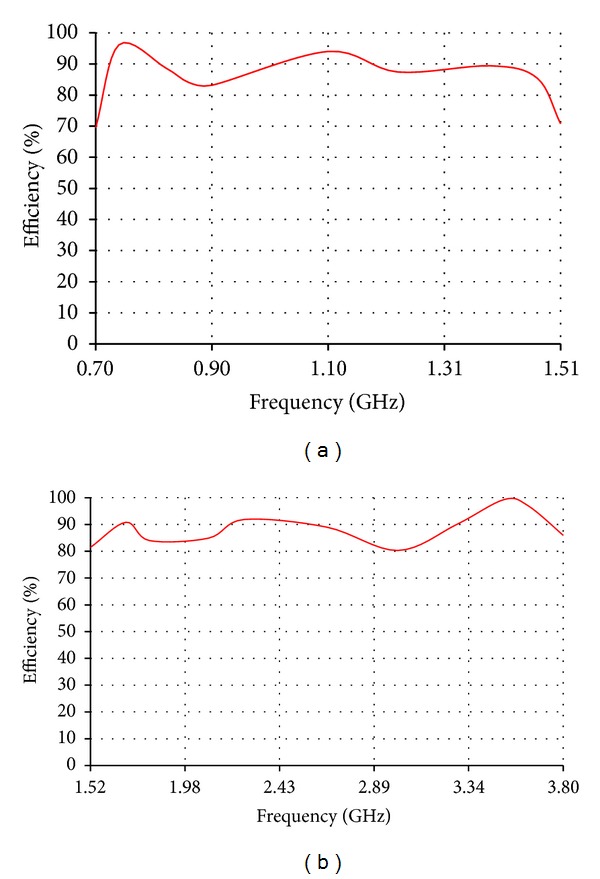
Efficiency of (a) the low band antenna and (b) the high band antenna.

**Figure 4:**
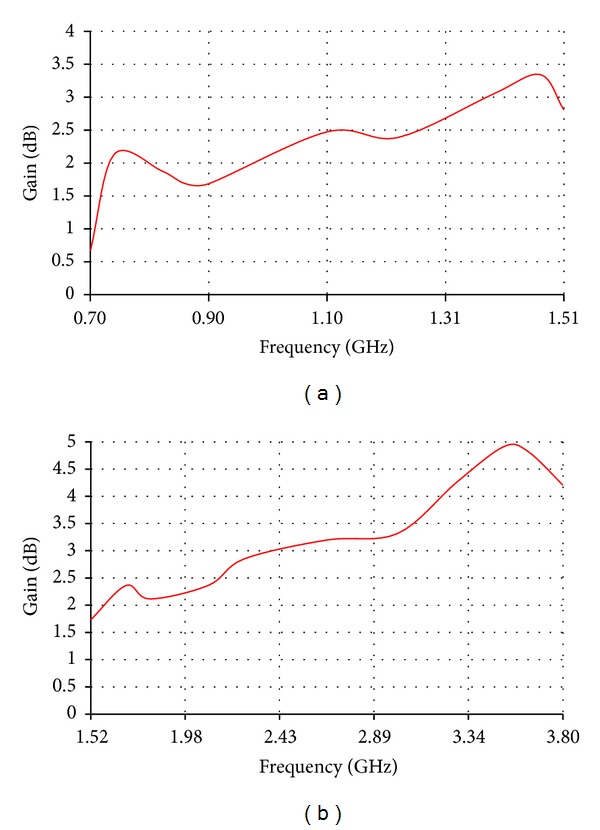
Gain of (a) the low band antenna and (b) the high band antenna.

**Figure 5:**
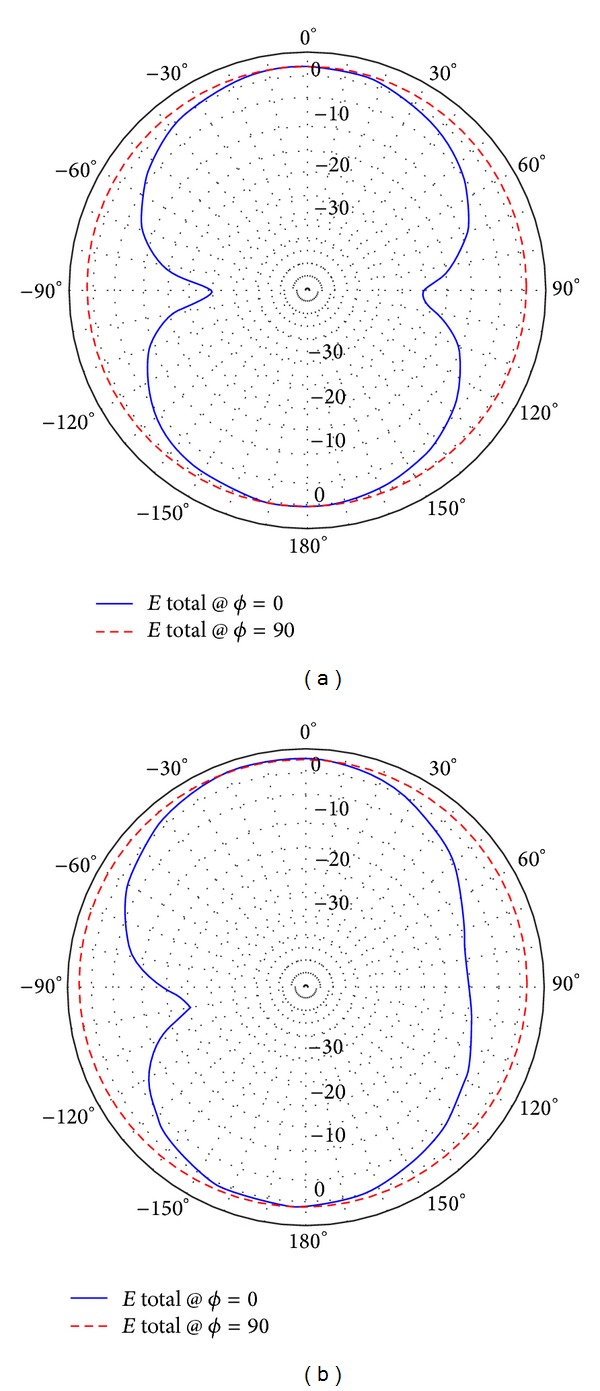
Radiation patterns of (a) the low band antenna at 900 MHz and (b) the high band antenna at 2.3 GHz.

The low band antenna has a volume of 1.3 × 0.4 × 14.5 = 7.54 cm^3^. It should be noted that the proposed volume is the total volume of the antenna because it does not require an additional ground plane or matching circuits. The high band antenna has a volume of 1.2 × 0.4 × 5.9 = 2.832 cm^3^. Like the low band antenna, the proposed volume of the high band antenna is the total volume of the antenna. As Figure 2(a) shows, the return loss of the low band antenna is lower than −8 dB over most of the bands having a maximum value of −5.5 dB. For the high band antenna, Figure 2(b) shows that the return loss is also lower than −8 dB all over the band. The total efficiency shown in Figures 3(a) and 3(b) demonstrates 80% and 90% average efficiencies of the low band and high band antennas, respectively. The gain of the low band antenna is shown in Figure 4(a) as a function of the frequency, while the high band antenna's gain is shown in Figure 4(b). The Radiation patterns of the low band and high band antennas are demonstrated in Figure 5(a) at 900 MHz and Figure 5(b) at 2.3 GHz, respectively, at phi = 0 and phi = 90.

### 2.2. Reduced Size Version of the Low Band and High Band Antennas

**Figure 6:**
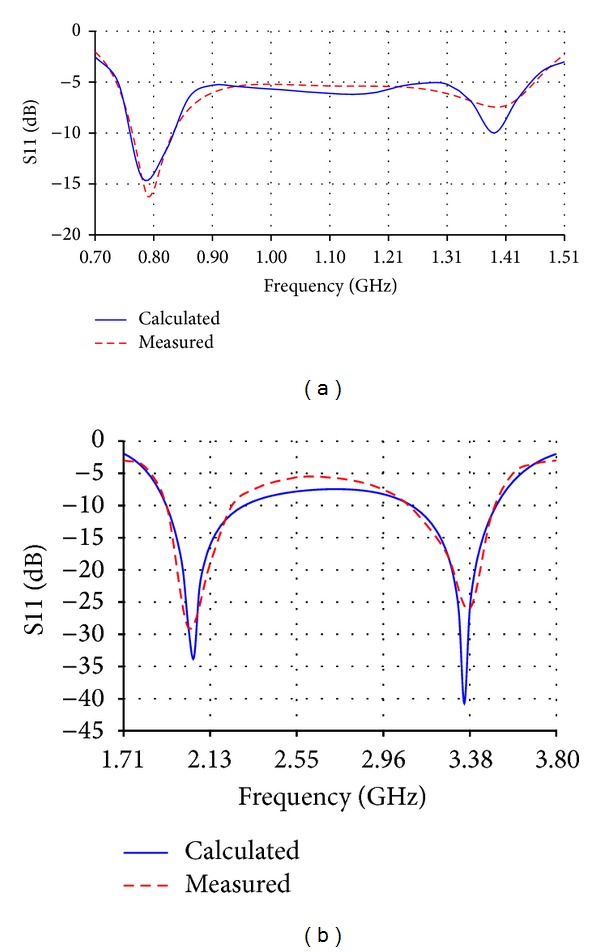
Return loss of (a) the reduced size version of the low band antenna and (b) the reduced size version of the high band antenna.

**Figure 7:**
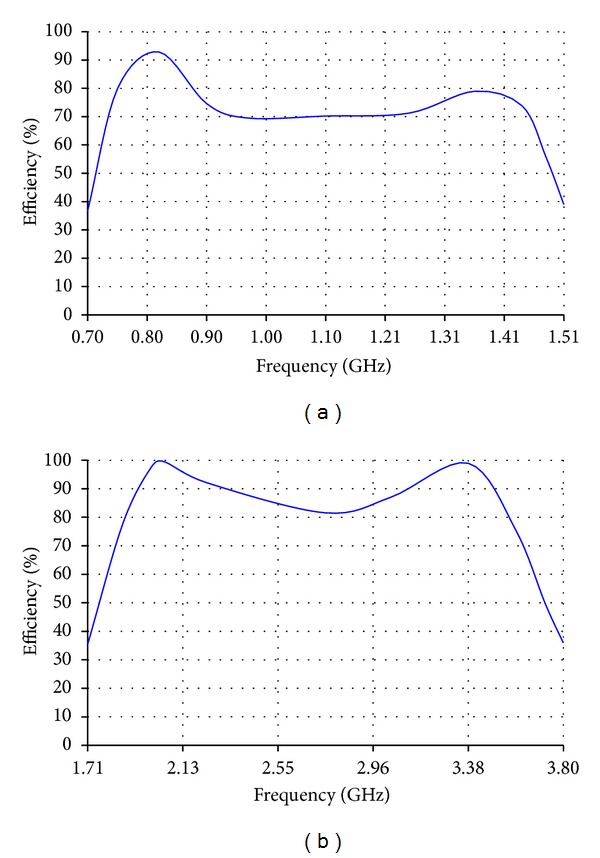
Efficiency of (a) the reduced size version of the low band antenna and (b) the reduced size version of the high band antenna.

**Figure 8:**
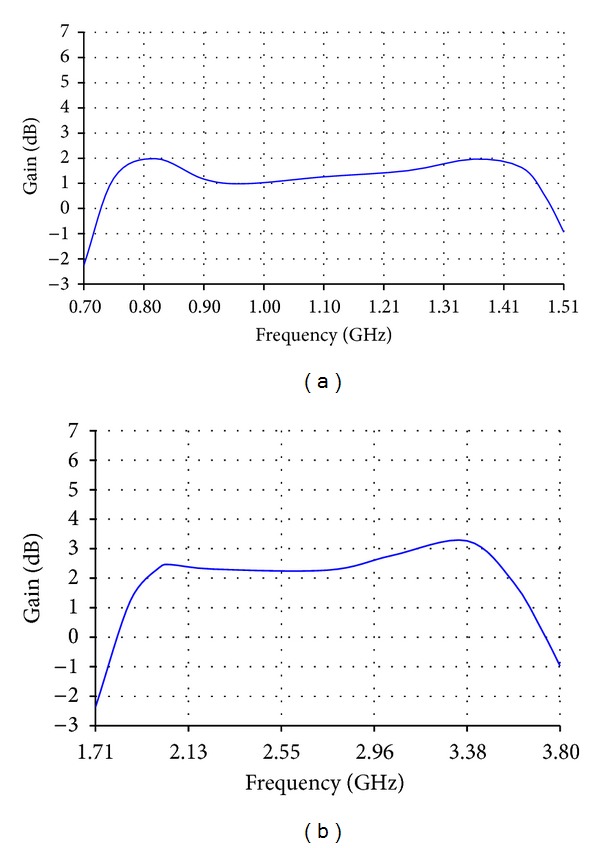
Gain of (a) the reduced size version of the low band antenna and (b) the reduced size version of the high band antenna.

**Figure 9:**
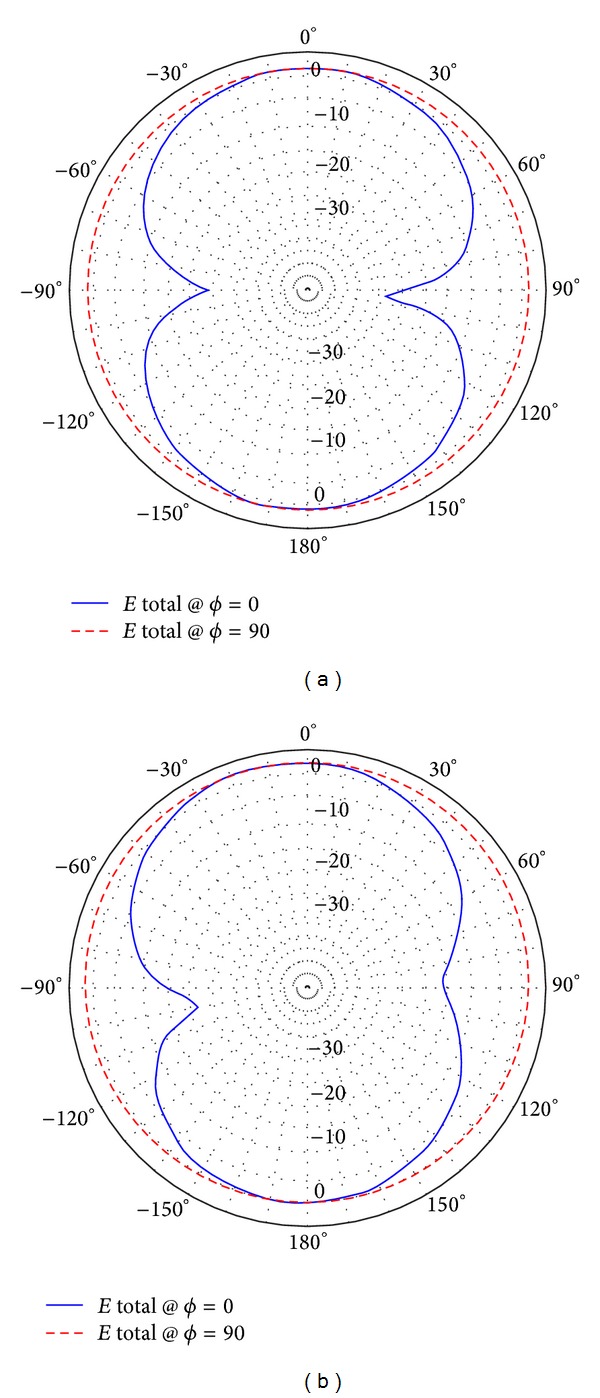
Radiation patterns of (a) the reduced size version of the low band antenna at 750 MHz and (b) the reduced size version of the high band antenna at 2.7 MHz.

The reduced size version of the low band antenna has a total volume of 0.4 × 0.4 × 15.6 = 2.496 cm^3^, while that of the high band antenna has a total volume of 0.4 × 0.4 × 5.9 = 0.944 cm^3^. As Figure 6(a) shows, the return loss of the low band antenna is lower than −5 dB almost all over the frequency band from 698 MHz to 1.51 GHz which is 73% bandwidth. For the reduced size version of the high band antenna, Figure 6(b) shows that most of the calculated and measured return losses is lower than −5 dB all over the frequency band from 1.71 GHz to 3.8 GHz which is 75.8%. The total efficiencies shown in Figures 7(a) and 7(b) demonstrate almost 70% and 80% average efficiencies of the reduced size versions of low band and high band antennas, respectively. The gain of the reduced size version of the low band antenna is higher than 1 dBi over most of the bands as shown in Figure 8(a), while the high band antenna's gain is more than 2 dBi as shown in Figure 8(b). The radiation patterns of the low band and high band antennas are presented in Figure 9(a) at 750 MHz and Figure 9(b) at 2.7 GHz, respectively.

## 3. MIMO Diversity Configuration

Multiples of low band and high band antennas can be used for LTE MIMO diversity coverage in laptops, tablets, and smart phones. Each of these different situations has been studied and will be presented.

### 3.1. MIMO Configurations for Smart Phones

**Figure 10:**
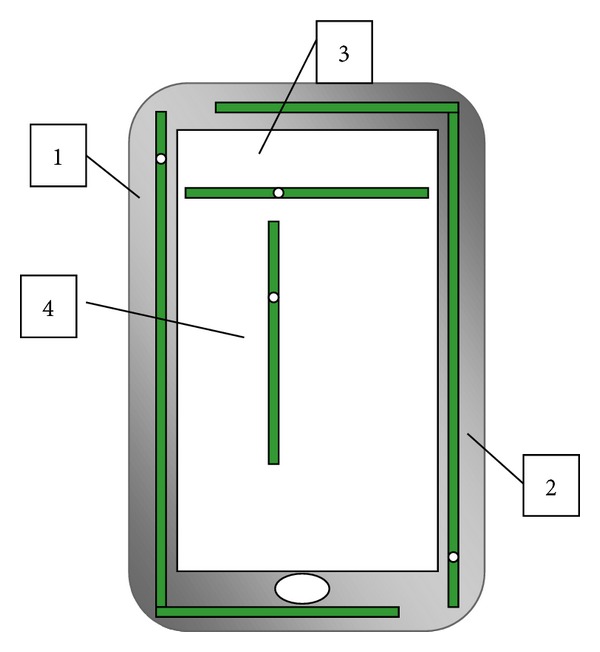
The schematic diagram of MIMO wideband antenna solution for LTE smart phones.

The new wideband antenna can be customized for implementation in smart phones. It can be bent or wrapped as an “L” shape to fit in the void around the chassis of the handset. This customization has been studied numerically and experimentally for 2 × 2 MIMO of the reduced size low band and high band antennas. As shown in the schematic in Figure 10, the primary reduced size low band antenna is marked as number “1” and the diversity antenna is marked as number “2.” The primary and diversity antennas are wrapped in “L-” shaped configurations. A 2 × 2 MIMO configuration of the reduced size high band antenna has been also tested numerically and experimentally and their relative positions to each other have been optimized for the implementation in smart phones. The relative positions of the primary and diversity antennas are shown in Figure 10. The primary reduced size high band antenna is marked as number “3” and the diversity antenna is marked as number “4.”

**Figure 11:**
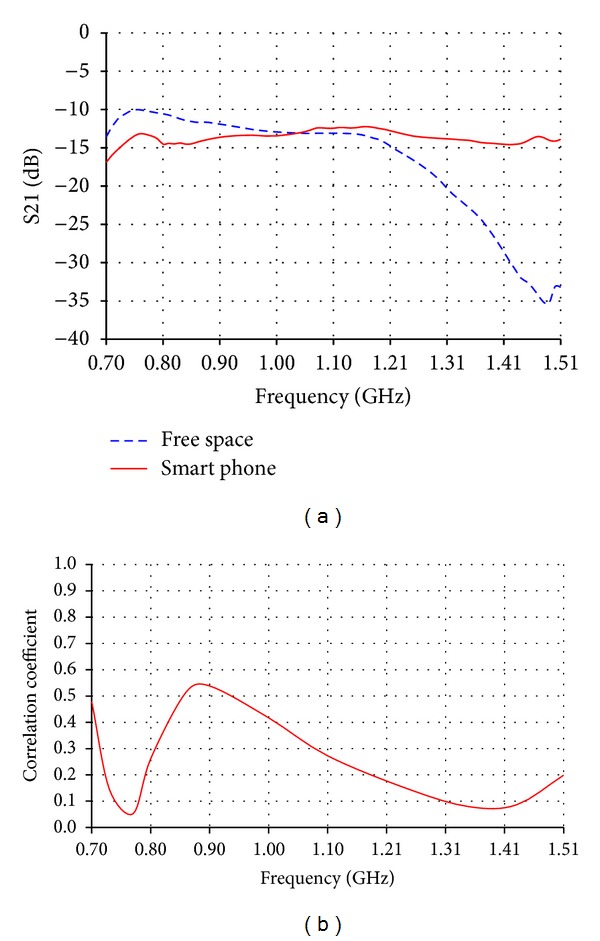
(a) Measured isolation (S21) and (b) correlation coefficient between primary and diversity MIMO low band antennas.

**Figure 12:**
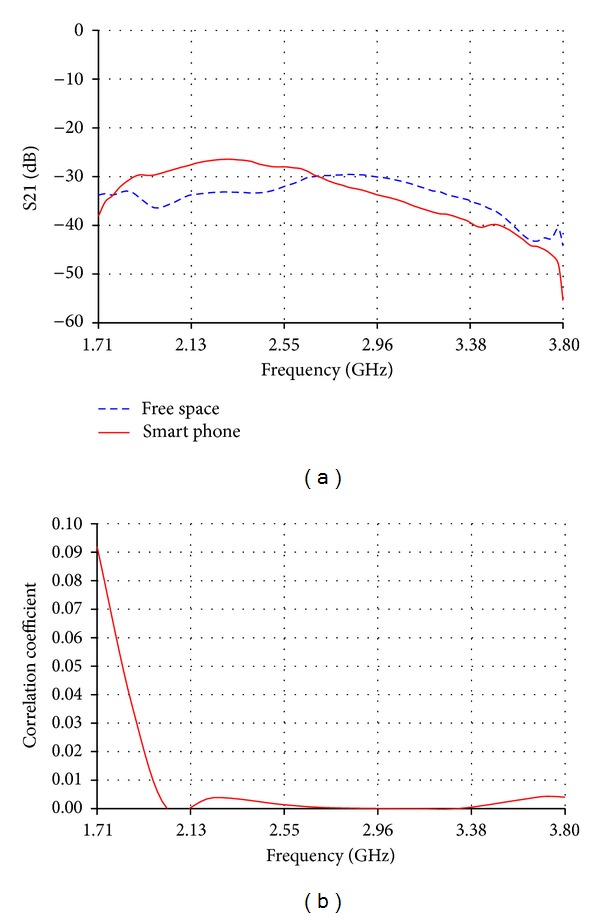
(a) Measured isolation (S21) and (b) correlation coefficient between primary and diversity MIMO high band antennas.

The measured isolation between the primary and diversity low band antennas has been investigated. As shown in Figure 11(a), acceptable isolation values, lower than −10 dB, are obtained from isolation measurements (S21) in free space. The measured isolation on the chassis of smart phone was much better than the isolation in free space, as it has an average of −15 dB over most of the bands. This is due to the fact that the phone is acting as an isolating medium which guarantees lower coupling between the primary and diversity antennas. The correlation coefficient between primary and diversity low band antennas was lower than 0.55 as shown in Figure 11(b). On the other hand, the measured isolation between the primary and diversity high band antennas was lower than −30 dB as shown in Figure 12(a). This is an expected isolation result due to the perpendicular position of the primary antenna relative to the diversity antenna which have been proved to be the best isolation technique in such a small available space inside handsets [9]. The perpendicular relative position also has a positive effect on the correlation coefficient. As shown in Figure 12(b), the maximum correlation value does not exceed 0.1 and most of the values are lower than 0.01.

**Figure 13:**
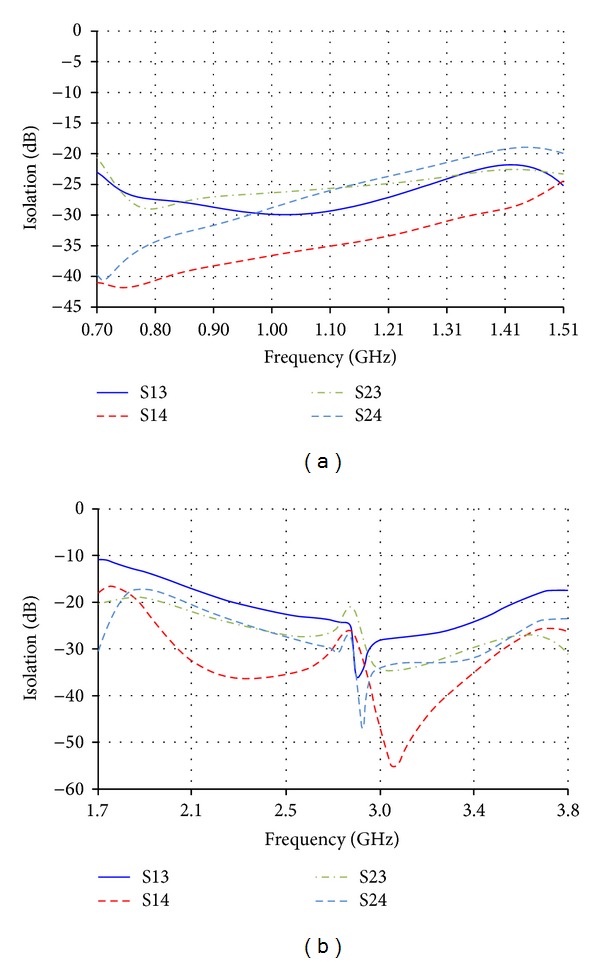
Isolation between each of low band and high band antennas in (a) the low frequency band and (b) the high frequency band on smart phones.

The urgent demand for universal LTE smart phones requires an antenna solution that is able to cover most of the LTE bands for global roaming. By combining the two new wideband antennas together in one device, it will fulfill that need as demonstrated in Figure 10. The high band antennas marked as numbers 3 and 4 are located in a higher plane above the low band antennas by 1 mm. The biggest concern in this case is the isolation values between the low band and high band antennas. The isolation is calculated for this case and the results are mostly lower than −20 dB for low band and high band frequency spectrums as shown in Figures 13(a) and 13(b), respectively.

### 3.2. MIMO Configurations on Laptops and Tablets

**Figure 14:**
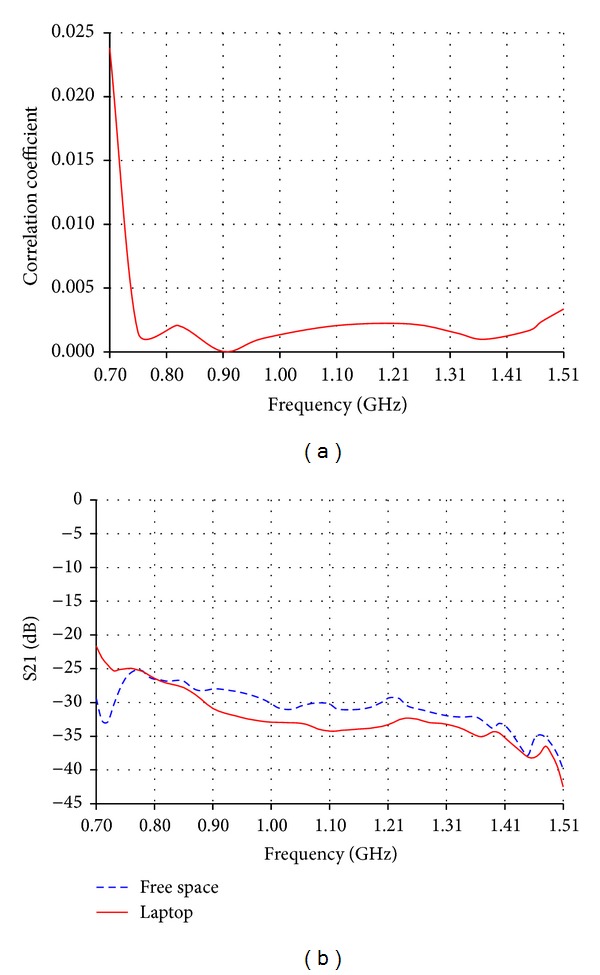
(a) Correlation coefficient and (b) measured isolation between primary and diversity MIMO low band antennas perpendicular to each other and 10 cm apart.

**Figure 15:**
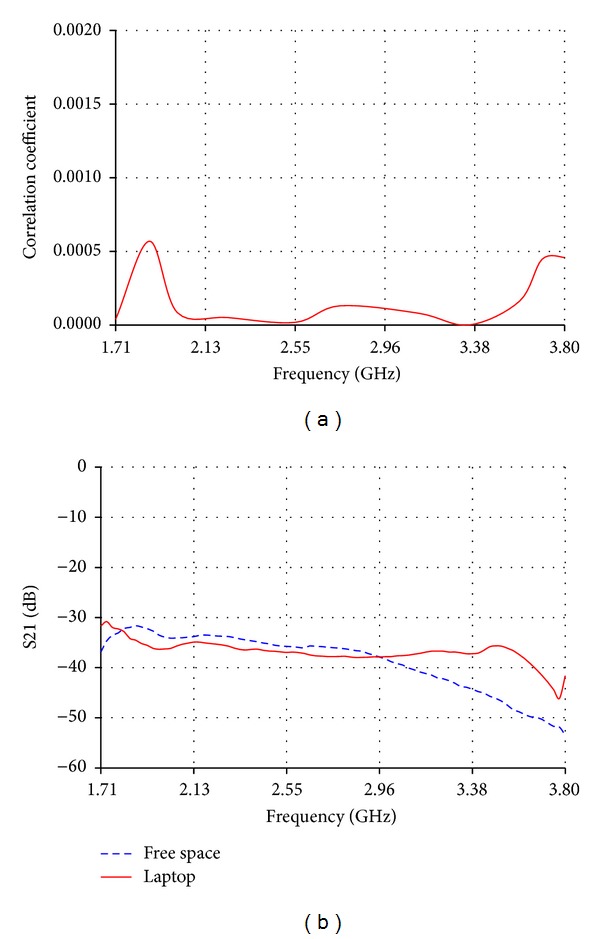
(a) Correlation coefficient and (b) measured isolation between primary and diversity MIMO high band antennas perpendicular to each other and 10 cm apart.

For 2 × 2 MIMO diversity, a primary antenna and a diversity antenna can be placed 10 cm apart and perpendicular to each other inside laptops and tablets. Although primary and diversity antennas are operating over the same frequency band at the same time, the coupling effect is significantly reduced as a result of antennas perpendicular relative positions [9]. Thus, this position will have a positive effect on the calculated correlation coefficient between the two of the reduced size low band antennas, as shown in Figure 14(a). The maximum correlation coefficient value is lower than 0.025. The isolation between the primary and the diversity antennas has been measured on a laptop and in free space keeping the same relative positions and distance between them. The measured isolation on the laptop gets improved over most of the bands as demonstrated in Figure 14(b). The previous experiments are repeated for 2 × 2 MIMO of the reduced size high band antennas perpendicular to each other and 10 cm apart. In terms of wavelength, the distance between the primary and the diversity antennas is larger in the high frequency band than in the low frequency band. This is reflected in a positive way on the correlation coefficient values which are lower than 0.001 as shown in Figure 15(a). Also a lower isolation than −30 dB in free space and on the laptop is shown in Figure 15(b).

## 4. Conclusion

A novel very wideband MIMO antenna solution was developed, capable of covering 39 LTE bands besides 2G and 3G. This provided device manufacturers with a new antenna technology to fulfill the urgent need for universal LTE smart phones, tablet computers, laptops, and notebooks.
